# The Influence of Plant Isoflavones Daidzein and Equol on Female Reproductive Processes

**DOI:** 10.3390/ph14040373

**Published:** 2021-04-17

**Authors:** Alexander V. Sirotkin, Saleh Hamad Alwasel, Abdel Halim Harrath

**Affiliations:** 1 Department of Zoology and Anthropology, Constantine the Philosopher University in Nitra, 949 01 Nitra, Slovakia; 2 Department of Zoology, College of Science, King Saud University, Riyadh 12372, Saudi Arabia; sawasel10@hotmail.com (S.H.A.); halim.harrath@gmail.com (A.H.H.)

**Keywords:** daidzein, equol, reproduction, ovarian folliculogenesis, oogenesis, proliferation, apoptosis, hormone, reception, fecundity

## Abstract

In this review, we explore the current literature on the influence of the plant isoflavone daidzein and its metabolite equol on animal and human physiological processes, with an emphasis on female reproduction including ovarian functions (the ovarian cycle; follicullo- and oogenesis), fundamental ovarian-cell functions (viability, proliferation, and apoptosis), the pituitary and ovarian endocrine regulators of these functions, and the possible intracellular mechanisms of daidzein action. Furthermore, we discuss the applicability of daidzein for the control of animal and human female reproductive processes, and how to make this application more efficient. The existing literature demonstrates the influence of daidzein and its metabolite equol on various nonreproductive and reproductive processes and their disorders. Daidzein and equol can both up- and downregulate the ovarian reception of gonadotropins, healthy and cancerous ovarian-cell proliferation, apoptosis, viability, ovarian growth, follicullo- and oogenesis, and follicular atresia. These effects could be mediated by daidzein and equol on hormone production and reception, reactive oxygen species, and intracellular regulators of proliferation and apoptosis. Both the stimulatory and the inhibitory effects of daidzein and equol could be useful for reproductive stimulation, the prevention and mitigation of cancer development, and the adverse effects of environmental stressors in reproductive biology and medicine.

## 1. Introduction

Reproduction is considered to be the most important process for the continued existence of species, but it is also a complicated process from a regulatory viewpoint. Understanding the physiological and natural regulators of reproduction is important for characterizing, predicting, and controlling reproductive processes, and for the prevention and treatment of reproductive disorders. A promising regulator, protector, and therapy is daidzein and its related plant molecules. In recent decades, daidzein was widely studied because of its health benefits, mainly in the prevention of cancer. The results of these epidemiological [1], biomedical [2,3,4,5], and biochemical [6,7,8] studies were described in several reviews. There are, however, no reviews summarizing the existing literature on daidzein and its effect on reproductive processes. Available reviews on daidzein in relation to female reproduction do not include findings from the last decade [9], or they only explore a niche area of the field, e.g., daidzein’s effect on preventing menopausal syndrome [10,11] or estrogen-related illnesses [12]. Our review incorporates recent findings on the influence of daidzein and its metabolite equol on animal and human female reproductive processes (ovarian cycle, follicullo- and oogenesis), basic ovarian-cell functions (viability, proliferation, apoptosis), the pituitary and ovarian endocrine regulators of these functions, the possible intracellular mechanisms of daidzein action on ovarian cells, controlling animal and human female reproductive processes, and how to make this application more efficient.

## 2. Provenance and Properties of Daidzein and Its Metabolite Equol

Isoflavones (including daidzein, the glycoside forms of daidzein, and glycitein, the methoxylated form of daidzein) are bioactive compounds that are present in significant quantities in legumes, soybeans, green beans, and mung beans. In grains (raw materials), they are mostly present as simple and complex O-glycosides, β-glucuronides and sulfate esters, and highly polar, water-soluble compounds. They are hardly absorbed by the intestinal epithelium and have insignificant biological activities. Beans are included in various food products owing to their digestibility, taste, bioavailability of nutrients, and higher amounts of bioactives. Processing steps include steaming, cooking, roasting, and microbial fermentation, which destroys protease inhibitors and cleaves the glycoside bond to yield absorbable and bioactive aglycone in the processed products, such as miso, natto, soy milk, tofu, and increases the product’s shelf life. Results are reliable (including the conversion of glucoside daidzein to aglicon daidzein, which hydrolyzes conjugated isoflavones with gut bacteria) [2,3,4,6]. Clinical studies show the differences between the biological activities of aglycone and conjugated forms of daidzein [2]. Processed soy food products were an integral part of diets in many Asia-Pacific countries for centuries, but in the last two decades, soy products were successfully integrated into Western diets because of their health benefits [3].

**Figure 1:**
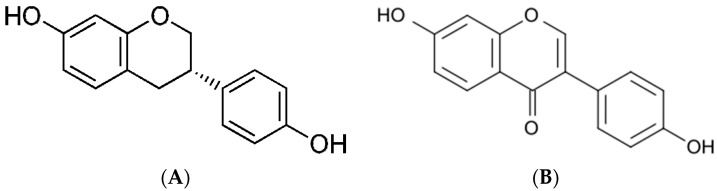
Chemical structures of (**A**) daidzein and (**B**) equol (https://pubchem.ncbi.nlm.nih.gov/ (accessed on 16 April 2021)).

Daidzein (7-hydroxy-3-(4-hydroxyphenyl)-4H-chromen-4-one) (PubChem ID 5281708, molecular formula C15H10O4; chemical structure indicated in Figure 1a, https://pubchem.ncbi.nlm.nih.gov/ (accessed on 16 April 2021)) is an isoflavonoid derived from L-phenylalanine. Daidzein is an aglycone form of daidzein. Its solubility in water is relatively low; therefore, for medical purposes, it is dissolved in alcohol or an alcohol/water mixture.

The primary biological and health effect of daidzein is because of its metabolite, equol ((3S)-3-(4-hydroxyphenyl)-7-chromanol) (PubChem ID, 6950272 molecular formula C15H14O3; chemical structure indicated in Figure 1b, https://pubchem.ncbi.nlm.nih.gov/ (accessed on 16 April 2021)). Equol is an isoflavandiol nonsteroidal estrogen and a secondary metabolite of daidzein that is the result of daidzein hydrolysis by gut bacteria. It is insoluble in water and acid-labile [5,13,14]. The conversion of daidzein into equol takes place in the intestine via the action of reductase enzymes belonging to incompletely characterized members of the gut microbiota. While all animal species analyzed so far produce equol, only between one-third and one-half of humans (depending on the community) can do so; ostensibly, those that harbor equol-producing microbes. Only 20%–30% of Westerners produce S-equol compared to 50%–70% of Asians; these subjects might be the only ones who can fully benefit from soy or isoflavone consumption [4,5,13]. Besides microflora, raw plant polyphenols can be metabolized by hepatocytes, intestines, and some other cells, including breast tumor cells. After daidzein enters the circulation via intestinal absorption, it may be transported to the liver, where it is conjugated by liver enzymes to form sulfated, glycosylated, and glucuronidated isoflavones, which are more water-soluble [2,3,6]. Additionally, inflammatory cells produce chemical oxidants that can react with polyphenols and inactivate them [6].

Although isoflavones are nonsteroidal, they have similarities in their chemical structure to that of mammalian estrogens, show estrogenic activities in biological assays, and induce estrogen-like effects in mammalian systems (see below).

Despite the low oral bioavailability, most polyphenols have significant biological effects that highlight the low bioavailability/high bioactivity paradox. This phenomenon can be explained by the high affinity and sensitivity of estrogen receptors, even to a low amount of phytoestrogen [8].

## 3. Mechanisms of Action of Daidzein and Its Metabolite Equol

Daidzein, like other flavanoid isoflavones, is a phenol plant compound that resembles vertebrate steroid hormones due to its molecular structure. Therefore, in vertebrates, it can bind steroid hormone receptors and exert estrogenic or antiestrogenic effects, thereby affecting physiological processes and illnesses dependent on steroid hormones [2,4,7,9,12]. As an antioxidant, daidzein can prevent oxidative stress and resulting disorders [6,7,8,14]. The manifestation of these illnesses could also be mitigated by daidzein due to its ability to promote antioxidant enzyme cytochromes such as P450 [14], which inhibits tyrosine kinase, a cell-proliferation promoter [7], and to activate the Akt signaling pathway, which promotes DNA fragmentation and cell apoptosis [15].

Equol, a daidzein metabolite with higher biological activity, can affect these processes via the direct binding of reactive oxygen species and the activation of antioxidative enzymes, binding to estrogen receptors, mitogen-activated protein kinase, protein kinase B (Akt), and epidermal growth factor receptor kinase and cyclin B/CDK complex (cell-proliferation promoters), transcription factor NFkB (promoter of inflammatory processes), nitric oxide-dependent intracellular signaling pathway, transcription factors FOXO3a and p53 (promoters of apoptosis), epigenetic mechanisms, including DNA methylation, histone modification, and microRNA regulation, and other intracellular signaling mechanisms [5].

## 4. Physiological and Therapeutic Actions of Daidzein and Its Metabolite Equol

Epidemiological, animal, and in vitro studies demonstrated the ability of daidzein to reduce the incidence of estrogen-dependent and aging-associated disorders, such as menopause, osteoporosis, cardiovascular diseases, cancer [1,2,3,4,6,7,8,11,14], cognitive disorders, and blood pressure [11]. Higher daidzein concentrations were associated with a lower risk of breast cancer and diabetes [1,7]. Daidzein impairs glucose and lipid metabolism and vascular inflammation associated with Type 2 diabetes [16]. It can promote the expression and activity of detoxifying enzymes [7], and prevent the toxic effects of polycyclic aromatic hydrocarbons [14]. Lastly, daidzein can assist with the treatment of viral infections, including COVID-19 [17].

Results of recent clinical trials and meta-analyses on the effects of equol demonstrated the preventive and curative action of equol on menopause, the cardiovascular system, bone health (prevention of osteoporosis), cancer, central-nervous-system functions, and mental disorders [5,13,15]. Equol may also modulate obesity and Type 2 diabetes by controlling the glycemic index, ameliorate chronic kidney disease, and prevent skin aging [5].

However, the effects of daidzein and equol depend on their income from food. For example, in East Asian countries, where the consumption of daidzein-containing products is 10 times higher than that in the West, the incidence of cardiovascular disease, osteoporosis, mental disorders, certain types of cancer, and menopausal symptoms is several times lower than that of the West [5]. The additional consumption of soy, daidzein, and equol did not affect the expression of menopausal symptoms in Chinese women [10]. 

Nevertheless, most clinical studies involving isoflavones suffered from small sample sizes, short trial durations, a lack of appropriate controls, the use of isoflavones from various sources, supplements with different aglycone contents, and other methodological flaws. Therefore, both specialists and regulatory agencies concluded that there is still no scientifically sound evidence of isoflavones reducing the risks and symptoms of any disease [5].

Lastly, isoflavones, including daidzein and its metabolites, may also be considered endocrine disruptors with possible negative health effects on certain parts of the population or on the environment [4,5]. Both in vitro and animal studies reported that isoflavones can interfere with different checkpoints of the hypothalamic/pituitary/thyroid system. Further, the estrogenic activity of isoflavones could be a hazard by promoting certain types of tumors; however, evidence for harm is also inconclusive [5].

## 5. Effects of Daidzein and Its Metabolite Equol on Female Reproductive Processes

Several laboratories studied the character of daidzein’s influence on animal reproductive systems and fecundity, but results were variable. Lamartiniere et al. [18] failed to find any effect of daidzein on rat ovarian histomorphology, weight, and fertility, the number of male and female offspring, or anogenital distances. Kaludjerovic et al. [19] did not find an influence with dietary daidzein on mice ovarian weight, the number of ovarian follicles, the number of multiple oocyte follicles, or the percentage of ovarian cysts; however, they observed the adverse effect of daidzein on ovarian structure and its ability to reduce the number of ovarian corpus lutea. In rats, daidzein suppressed follicular growth (reduced ovarian weight), but not ovarian folliculogenesis and fecundity (number of corpora lutea) or sexual behavior (lordosis quotient) [20,21]. Talsness et al. [22] reported the inhibitory influence of daidzein on rat ovarian folliculogenesis, which increased follicular atresia; reduced secondary and tertiary follicle numbers, and probably ovarian surface epithelium proliferation; induced cyst formation; and prolonged estrous. The ability to suppress growth and to induce the atresia of murine cultured ovarian follicles was also reported for quercetin metabolite equol [23].

Other studies showed the stimulatory action of daidzein on chicken ovarian germ cells [24], chicken ovarian folliculogenesis, and the ovulation/egg-laying rate [25]. The daidzein-induced promotion of rat ovarian folliculogenesis and a reduction in ovarian follicular atresia were observed by Medigović et al. [26]. Dorward et al. [27] reported that feeding mice a mixture of daidzein and genistein promoted ovarian and uterine growth, and increased the incidence of ovarian tumorigenesis.

The influence of daidzein on nonovarian reproductive organs remains unknown. Dietary daidzein did not affect uterine morphology and weight in rats [18] and mice [19]. No influence of daidzein on the proliferation of murine endometrium and endometrioma cells was found [28]. Kaludjerovic et al. [19] reported that dietary daidzein induced hyperplasia in the murine oviduct and abnormal histomorphological changes in the subjects’ uteri. 

Therefore, available information concerning the expression and characterization of daidzein action is inconsistent, although most relevant papers suggest inhibitory action with this polyphenol on rodent and chicken reproductive systems. It remains unknown whether daidzein can influence the human reproductive system. 

Daidzein did not affect viability in cultured bovine ovarian granulosa and luteal cells [29]. In some of our experiments [30], daidzein promoted cell viability and proliferation but not apoptosis by cultured porcine ovarian granulosa cells. In other experiments [31], daidzein reduced the viability of these cells, but this effect was not associated with changes in cell proliferation or apoptosis. Daidzein was able to induce the death of ovarian cancer cells both in vivo [32] and in vitro [32,33].

Therefore, daidzein can induce the death of cancer cells, but available data concerning its action on healthy ovarian cells’ viability, proliferation, and apoptosis remain inconclusive.

## 6. Hormonal Mechanisms of Daidzein and Equol Effects on Female Reproductive Processes

Female reproductive functions, like other functions, are controlled by a hierarchical system of exogenous and endogenous regulators—regulatory molecules produced in the hypothalamus and other areas of the central nervous system (CNS), pituitary hormones (mainly gonadotropins), hormones produced by the ovaries and other peripheral organs, receptors to hormones, and intracellular mediators of their action—and enzymes including protein kinases, transcription factors regulating cell proliferation, apoptosis, viability, and differentiation [34]. The influence of daidzein on some (but not all) of these regulatory molecules is documented.

In the available literature, we failed to find any evidence for daidzein action on the CNS. Furthermore, histomorphological studies did not reveal daidzein influence on morphophysiological indexes of activity in pituitary gonadotropes and lactotropes [26], indicating that daidzein does not affect gonadotropin- and prolactin-producing pituitary cells.

Feeding with daidzein increased the number of follicle stimulating hormone (FSH) and luteinizing hormone (LH) receptors in chicken ovarian follicles [25]. Furthermore, daidzein promoted the stimulatory action of LH on mouse ovarian-cancer development [27]. These observations indicate that daidzein can promote the reception and effect of gonadotropins on ovarian functions. The ability of daidzein to promote the generation of gonadotropin receptors, which was associated with an increase in ovarian folliculogenesis and the egg-laying rate [25] mentioned above, suggests that daidzein promotes ovarian-cell function through the upregulation of gonadotropin receptors. The exact mechanism remains unknown; however, estrogen via estrogen receptors can boost the production of gonadotropin receptors in the ovary [34]. Daidzein can promote the generation of estrogen receptors (see below) [35,36], and mimic gonadotropin action on ovarian cell proliferation [24]. This indicates that daidzein can promote the production of gonadotropin reception through the activation of estrogen receptors. Nevertheless, this hypothesis requires experimental validation.

The action of daidzein on peripheral hormones, including hormones of ovarian origin regulating reproductive functions, is well-documented, although substantial differences are evident in the character of daidzein action, even in similar experiments.

In the in vivo experiments of Medigović et al. [26], injections of diosgenin increased progesterone and estradiol, and decreased testosterone levels in rat serum. On the other hand, in the in vivo experiments of Lamartiniere et al. [18], feeding rats with daidzein reduced the concentration of circulating progesterone but not estrogen. 

Some in vitro experiments demonstrated a direct inhibitory action of daidzein and its metabolite equol on steroid hormones‘ release by ovarian cells. Equol inhibited the expression of steroidogenic enzymes, and the synthesis and release of progesterone, testosterone, androstenedione, and estradiol by cultured murine ovarian follicles [23]. Similarly, there was an inhibitory influence with daidzein on progesterone, but not the estradiol release of cultured porcine ovarian granulosa cells, as observed in the in vitro experiments of Nynca et al. [34,35]. Similar experiments by Sirotkin et al. [31] failed to detect daidzein action on progesterone and estradiol release by cultured porcine granulosa cells, but in these experiments, daidzein promoted testosterone output. Other experiments on porcine granulosa cells showed the upregulation of progesterone, but not testosterone and estradiol production [30]. Mlynarczuk et al. [29] did not observe any influence of daidzein on progesterone and estradiol release by cultured bovine granulosa and luteal cells. In their experiments, daidzein was a potent stimulator of enzymes responsible for oxytocin synthesis and release. 

Epidemiological studies [37] showed an association between decreased serum daidzein levels and decreased female serum adiponectin levels, and increased serum insulin levels, which could be associated with an elevated risk of ovarian cancer.

The similarity of daidzein and estrogen effects on rat ovarian folliculogenesis [22] and steroidogenesis [26] suggests there is a similar mechanism of action with these molecules on adult ovarian functions via estrogen receptors. This hypothesis was confirmed by the ability of both daidzein and estradiol to bind to estrogen receptors in ovarian and nonovarian tissue [2,3], and to upregulate the expression of estrogen receptors alpha and beta in porcine ovarian cells [35,36]. Moreover, the blockage of estrogen receptors prevented daidzein action on the proliferation of chicken ovarian germ cells [24]. On the contrary, there are reports [27,38] that daidzein can antagonize estrogen action on ovarian-cancer cells. Reports indicate that daidzein affects ovarian-cancer development via both the up- and the downregulation of estrogen receptors. The current literature does not contain direct evidence concerning daidzein action on and via receptors to other peripheral hormones regulating female reproductive processes, but such action cannot be excluded. 

Taken together, available data demonstrate the ability of daidzein and equol to regulate the release of steroid and nonsteroid hormones (progestogen, androgens, estrogens, oxytocin, adipokines), and to generate the production of gonadotropin and estrogen receptors. These signaling molecules could be constituents of the integrated endocrine pituitary–gonadal axis. For example, the production of progestogens, androgens, estrogens, and oxytocin is regulated by gonadotropins, while there is also the mutual stimulation of steroid hormones and oxytocin. The gonadotropin–ovarian hormone axis and receptors to these hormones are known regulators of basic ovarian functions and fecundity [34]. 

Therefore, available data demonstrate that daidzein can affect female reproductive processes via the system of extracellular self-stimulating hormones of pituitary, ovarian, and nonovarian origins and of their receptors. On the other hand, the involvement of only gonadotropin and estrogen receptors in mediating daidzein action is not yet indicated. 

## 7. Intracellular Mechanisms of Daidzein and Equol Effects on Female Reproductive Processes

Several studies outlined the possible intracellular mechanisms of daidzein action on ovarian cells. In vitro study results demonstrated that daidzein can suppress ovarian-cancer cell functions. This effect was associated with changes in the production or expression of regulators of cell proliferation (mitogen-activated protein kinases, cyclin B1) and of apoptosis (p-FAK, p-PI3K, p-AKT, p-GSK3β, and p21) [38,39]. This association indicates that these intracellular signaling molecules and the related signaling pathways could be involved in mediating daidzein action on ovarian-cancer cells. Nevertheless, it does not provide strong evidence for a mediatory role with these molecules and their functional interrelationships. The experiments of Mahalingam et al. [23] demonstrated that equol can inhibit growth and promote the atresia of cultured murine ovarian follicles, and that this action was associated with the reduced expression of the suppressor of cytoplasmic apoptosis, B-cell leukemia/lymphoma 2 (bcl-2), and increased expression of the promoter of this apoptosis, bcl2-associated X protein (bax) [23]. Therefore, these markers and regulators of apoptosis can also mediate the action of daidzein metabolite equol on healthy ovarian cells. 

The next mechanism of daidzein action on ovarian cells could be its antioxidative effect, with its ability to activate antioxidant enzymes, and block reactive oxygen species and their adverse effects on chicken ovarian germ cells [24] and rat ovaries [26]. Moreover, these experiments indicated that daidzein can affect chicken and rat cells by using two (estrogenic and antioxidant) mechanisms at once. The activation of both mechanisms has similar consequences: the suppression of ovarian-cell apoptosis and the promotion of their proliferation and viability, and thereby of ovarian folliculogenesis, oogenesis, and fecundity. Therefore, the mutual influence or functional inter-relationships between the estrogenic and antioxidant mechanisms of daidzein and equol cannot be excluded, although no strong experimental evidence for such inter-relationships and its mediators exists. 

## 8. Application of Daidzein and Equol in Reproductive Biology and Medicine

Available information concerning daidzein’s effect on reproductive processes suggests possible areas for application. The experiment results of Liu and Zhang [25] demonstrated the stimulatory action of daidzein on chicken ovarian follicle development and ovulation, and thereby the applicability of daidzein as a biostimulator of poultry egg-laying rates. The stimulatory action of daidzein on porcine ovarian-cell viability and proliferation [30], on rat ovarian folliculogenesis and uterine functions, and its ability to prevent atresia of rat and mouse ovarian follicles [26,27] indicates that daidzein can also be a stimulator of female reproductive processes in mammals. If daidzein can promote the activation of gonadotropin receptors in both chickens and mammalian ovaries, daidzein could be useful as a gonadotropin action enhancer in gonadotropin-induced ovulation in animal production and assisted reproduction. Furthermore, the ability of daidzein to promote the release of ovarian estradiol [26] and to activate estrogen receptors [2,3,35,36] suggests that daidzein or daidzein-containing food could be beneficial for the prevention of disorders induced by a deficit of estrogens or their receptors; e.g., reproductive aging and the other age-related disorders listed above.

On the other hand, numerous other data sources demonstrated the ability of daidzein [21,22] and equol [23] to suppress functions of the ovaries and of healthy ovarian cells [31]. These data suggest that the overconsumption of daidzein or daidzein-containing food could have adverse effects on animal and human reproductive processes. Additionally, the ability of daidzein to suppress ovarian-cancer cell functions demonstrates its applicability for the prevention and mitigation of ovarian-cancer development [32,33]. The antioxidant effects of daidzein indicate its potential applicability for the prevention of both ovarian cancer and other reproductive disorders induced by oxidative stress, and for an increase in the reproductive system’s resistance to stress-inducing environmental factors. For example, daidzein was able to prevent the adverse effects of polychlorinated biphenyls on oxytocin release by cultured bovine ovarian cells, and on uterine-cell contractions and prostaglandin F release [40]. 

Therefore, the stimulatory, inhibitory, and protective effects of daidzein on female reproductive processes could principally be applicable in animal production, human reproductive biology, and human and veterinary medicine. 

## 9. Conclusion and Possible Direction of Further Studies

**Figure 2:**
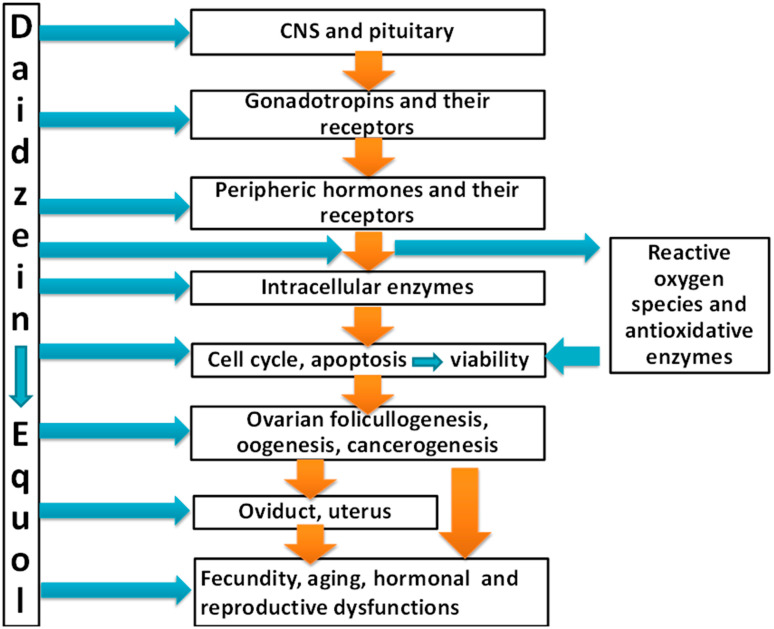
Targets and mechanisms of action of daidzein and equol on female reproductive processes and their dysfunctions.

The available literature demonstrates the influence of daidzein and its metabolite equol on various nonreproductive and reproductive processes, and their disorders. Daidzein and equol can both up- and downregulate ovarian reception of gonadotropins, healthy and cancerous ovarian-cell proliferation, apoptosis, viability, ovarian growth, follicullo- and oogenesis, and follicular atresia. These effects could be mediated by daidzein and equol on hormonal production and reception, reactive oxygen species, and intracellular regulators of proliferation and apoptosis. The targets and mechanisms of daidzein and equol action on the female reproductive system are summarized in Figure 2. 

Both the stimulatory and inhibitory effects of daidzein and equol could be useful for reproductive stimulation, and for the prevention and mitigation of cancer development and the adverse effects of environmental stressors in reproductive biology and medicine. 

Nevertheless, data concerning daidzein effects are contradictory, and data concerning equol are insufficient to generate any general conclusions concerning the character of the effect of these molecules on female reproduction. The causes of such differences in daidzein action observed in various experiments remain unknown. The main data were obtained from in vitro experiments, the results of which require validation by corresponding in vivo studies. The in vivo studies were performed mainly on laboratory animals, while only a few human epidemiological studies were performed. More is known about daidzein’s influence on cancer than on healthy female reproductive processes. It remains unknown whether daidzein can be an endocrine disrupter that jeopardizes reproduction [5]. Therefore, understanding the application of daidzein and its metabolites’ action on female reproductive processes requires further study.

Furthermore, the application of daidzein and daidzein-containing food is limited by the low bioavailability of this isoflavone. To address this, numerous promising strategies, such as the use of an absorption enhancer, structural transformation (e.g., prodrugs, glycosylation), and pharmaceutical technologies (e.g., carrier complexes, nanotechnology, cocrystals) were developed and applied to deliver poorly water-soluble flavonoids [41]. Nevertheless, the problem of enhancing the bioavailability and efficiency of daidzein awaits a solution. 

Overall, daidzein and its metabolites could be an efficient tool to control female reproductive processes, including fecundity, and in the prevention and treatment of reproductive disorders. Our current knowledge is insufficient to adequately understand and use these flavonoids and their effects on female reproductive events. They represent an important subject for future basic and applied research.

## Data Availability

No new data were created or analyzed in this study. Data sharing is not applicable to this article.
